# Regulatory reforms and the impact on overseas new drug accessibility in China: an analysis from 2010 to 2025

**DOI:** 10.3389/fphar.2026.1961582

**Published:** 2026-10-16

**Authors:** Yaoyang Tang, Li Yang

**Affiliations:** School of Business Administration, Shenyang Pharmaceutical University, Shenyang, China

**Keywords:** accessibility, China, overseas new drugs, R&D strategies, regulatory reforms, reimbursement access

## Abstract

In recent years, China has undertaken a series of regulatory reforms aimed at enhancing the accessibility of overseas new drugs by mitigating marketing approval lag and expediting their inclusion in the National Reimbursement Drug List (NRDL). This study conducted a retrospective analysis of overseas new drugs approved in China between 2010 and 2025, emphasizing temporal shifts in drug characteristics, regulatory efficiency, and approval lag across different reform periods. Furthermore, the study examined the reimbursement status of overseas new drugs in China, identified factors influencing their inclusion in the NRDL, and explored the China-oriented research and development (R&D) strategies adopted by their holders. Following the implementation of these reforms, the number of overseas new drug approvals increased significantly, with the cumulative total reaching 361 over the 2010–2025 study period. Simultaneously, the approval lag of overseas new drugs in China experienced a reduction, with the median duration decreasing from 1,442 to 1,188 days, using the United States as a reference. Notable improvements were observed in reimbursement access, as 213 out of 361 drugs (59.0%) were incorporated into the NRDL, and the median time for inclusion in the NRDLs was reduced from 2,297 to 743 days. However, we found that drugs approved after the reforms did not have a significantly higher likelihood of being included in the NRDL. Analyses specific to different R&D strategies indicated greater regulatory flexibility in China’s acceptance of overseas clinical evidence. Notably, drugs participating in multi-regional clinical trials (MRCTs) were associated with the shortest median approval lag, measured at 737.5 days. Overall, these findings highlighted the critical importance of China’s integration into global drug development initiatives and the need for enhanced coordination between regulatory and reimbursement processes.

## 1 Introduction

The prompt and efficient access of patients to new drugs is contingent upon streamlined regulatory approval and reimbursement processes (Zhu and Zhang, 2025; Wang et al., 2025). This issue is particularly pertinent to overseas new drugs, which have historically been instrumental in meeting unfulfilled clinical needs in China (Mao et al., 2025), but have encountered obstacles at both stages of the process (Su et al., 2023; Liu G. G. et al., 2022). Several factors have collectively contributed to the approval lag of overseas new drugs in China, including the limited regulatory acceptance of foreign clinical evidence, China’s delayed participation in multi-regional clinical trials (MRCTs), and the requirement for China-specific clinical data (Jiang et al., 2025; Du et al., 2025; Zhu and Chen, 2024). Additionally, infrequent updates to the NRDL and protracted adjustment procedures have further hindered reimbursement access (Liu G. G. et al., 2022).

In response to these enduring challenges, China implemented a series of regulatory and reimbursement reforms. Notably, in 2017, the NMPA became a member of the International Council for Harmonisation of Technical Requirements for Pharmaceuticals for Human Use (ICH), signifying a critical advancement towards harmonizing its drug registration requirements with international standards (Liu Y. et al., 2022). Subsequent initiatives permitted the utilization of data generated from MRCTs to support marketing applications (China Food and Drug Administration, 2017), expedited the review process for Investigational New Drug (IND) applications (National Medical Products Administration, 2024), and established criteria for the regulatory acceptance of overseas clinical trial data (Center for Drug Evaluation and National Medical Products Administration, 2018). Furthermore, the introduction of a dedicated expedited pathway for drugs included in the Urgently Needed Overseas Drugs (UNOD) lists (National Medical Products Administration and National Health Commission, 2018), published by the National Medical Products Administration (NMPA) between 2018 and 2020, underscored China’s focused efforts to mitigate approval lag for overseas new drugs (Wei et al., 2025). Concurrently, the establishment of the National Healthcare Security Administration (NHSA) in 2018 centralized national responsibilities related to the pricing, reimbursement, and management of the NRDL. Subsequent reimbursement reforms institutionalized annual updates to the NRDL and established price negotiation mechanisms, as well as a manufacturer-initiated application process for NRDL inclusion (Liu G. G. et al., 2022; Macabeo et al., 2023). A comprehensive overview of additional policies, regulations, and technical guidance is provided in Supplementary Table S1. In the wake of these reforms, there has been an increase in the approval of overseas new drugs in China in recent years. Although prior studies have examined the impact of these reforms on overseas drug access in China, the majority have concentrated on the period before 2022 (Mao et al., 2025; Su et al., 2023; Peng and Zheng, 2023; Zhu and Chen, 2023), leaving a paucity of evidence regarding the long-term evolution of reimbursement access. As a result, there is a notable gap in research that offers a comprehensive assessment of the long-term changes in both regulatory approval and reimbursement access for overseas new drugs in China.

To address this gap, this study conducted a comprehensive assessment of changes in drug characteristics, regulatory efficiency, approval lag, and reimbursement access from 2010 to 2025. It further explored post-reform R&D strategies and their relationship with approval lag. By integrating clinical development, regulatory approval, and reimbursement into a unified framework, this study transcended stage-specific analyses to offer a holistic, long-term evaluation of whether enhancements in regulatory efficiency had been paralleled by improvements in reimbursement access. This approach elucidated both advancements and deficiencies throughout the entire access pathway for overseas new drugs in China.

## 2 Materials and methods

### 2.1 Data source and study sample

The study sample comprised overseas new drugs receiving their first marketing approval in mainland China between 1 January 2010, and 31 December 2025. In this research, overseas new drugs were defined as therapeutic medicinal products containing chemical or biological active ingredients that had either been marketed or submitted for marketing authorization outside mainland China (excluding Hong Kong SAR, Macao SAR, and the Taiwan region) but had not yet received marketing approval in mainland China. In this study, the classification of a drug as either an “overseas innovative drug” or an “overseas original drug” was based on the global marketing status of its active ingredient at the time of submission of its initial marketing application to the NMPA. Drugs whose active ingredients had not received marketing approval overseas by the NMPA submission date were categorized as overseas innovative drugs, including those with pending overseas marketing applications. Conversely, drugs whose active ingredients had been approved for overseas marketing prior to the submission date were designated as overseas original drugs. It is important to note that any overseas approvals granted subsequent to the NMPA submission date did not affect the initial classification. Each drug with an approved initial indication was treated as a separate analytical unit, referred to as an “overseas new drug item.” Accordingly, the final analytical sample comprised 361 overseas new drugs.

We searched drug review reports published by the NMPA (National Medical Products Administration, 2025), as well as the YAOZHI database (YAOZHI, 2025), a widely used commercial pharmaceutical database in China. For each drug, essential data were extracted, including the drug class, Anatomical Therapeutic Chemical (ATC) classification, origin, dosage form, indication, target population, approval date in China, and any designated expedited programs (Breakthrough Therapy Designation, Priority Review, or Conditional approval). For drugs approved between 2018 and 2025, additional information was gathered concerning pivotal clinical evidence and R&D strategies. The limited public availability of review reports for drugs approved between 2010 and 2017 impeded the systematic retrieval of the clinical information necessary to apply consistent classification criteria. As a result, the analysis of R&D strategies was confined to the 279 drugs approved between 2018 and 2025, and no cross-period comparison was conducted. Generic drugs, supplemental applications, overseas modified drugs, *in vitro* diagnostics (IVDs), radiopharmaceuticals, therapeutics for COVID-19 and vaccines were excluded from this study. Supplementary Figure S1 summarizes drug selection and analytical sample derivation.

The origin of each drug was identified as the country in which the manufacturer was based. Utilizing rare disease catalogs published by the Chinese government, we assessed whether each drug qualified as a rare disease drug. The classification of a drug as a UNOD was determined according to the lists issued by the NMPA. Drugs appearing on these lists were eligible for China’s approval process for UNODs (refer to Supplementary Table S2). To ascertain the inclusion and timing of each drug in the NRDL, this study examined all editions of the NRDL published during the study period, including the most recent edition, effective 1 January 2026. Consequently, the NRDL inclusion status was evaluated as of the fixed cutoff date of 1 January 2026. Drugs listed in at least one NRDL edition by this date were classified as “included”, while all other drugs were classified as “not included” by the cutoff date. This fixed-cutoff definition was consistently applied as the outcome definition for primary analyses of NRDL inclusion status throughout the study. The United States served as the benchmark for measuring marketing approval lag. For each overseas new drug approved in China, the corresponding approval date from the U.S. Food and Drug Administration (FDA) was extracted from FDA review reports (U.S. Food and Drug Administration, 2025). Only overseas new drugs with corresponding FDA approval records were included in the analysis of approval lag.

### 2.2 Definitions

Approval lag was defined as the interval between FDA marketing approval and NMPA marketing approval (Xu et al., 2026; Luo et al., 2023a). Negative lag values were retained and interpreted as indicating approval in China preceding approval in the comparator jurisdiction. The review time was calculated as the interval from the acceptance date of the initial marketing application to the NMPA approval date (Zhu and Liu, 2023). Time to reimbursement was calculated from the date of NMPA approval to the date of first inclusion in the NRDL (Wang et al., 2025).

In this study, “Strategy A” was characterized by exclusive reliance on international clinical data, exempting the need for domestic clinical trials. “Strategy B” involved participation in MRCTs that included China, with the resultant evidence being directly utilized to support marketing approval within the country. “Strategy C” entailed the use of international clinical data as the primary evidence, supplemented by a bridging study conducted in China. In contrast, “Strategy D” involved conducting additional clinical trials in China to generate pivotal evidence. Ethnic insensitivity was considered verified only when the Center for Drug Evaluation (CDE) in China explicitly indicated the absence of ethnic differences or concluded, based on pharmacokinetic (PK), pharmacodynamic (PD), efficacy, and safety data, that the findings were clinically consistent across Chinese and international or global populations without necessitating ethnicity-related adjustments to dosage or risk management. Conversely, ethnic insensitivity was deemed unverified when the evidence was insufficient or suggested potentially clinically significant ethnic differences. Drugs classified as “superior” were identified as those targeting serious or life-threatening conditions, rare diseases without effective treatments, or those exhibiting significant enhancements in efficacy or safety compared to existing therapies. The R&D strategy for each drug was assessed through a detailed, case-by-case analysis of the pivotal clinical trials as documented in the respective CDE review reports, and their evidentiary contribution to securing marketing approval in China. Supplementary Table S3 presents representative borderline cases along with the rationale for their classification.

### 2.3 Data quality and bias mitigation

Potential sources of bias encompassed incomplete historical coverage, potential selection bias in the approval-lag analysis due to missing corresponding FDA approval records, terminological and reporting inconsistencies across data sources, and potential misclassification of drug characteristics and R&D strategies. To mitigate these risks, one author initially retrieved and extracted the data, classifying the relevant variables, including R&D strategies, while the other author subsequently verified the extracted data and classifications against the original sources. For approval-lag analyses, FDA approval dates were cross-validated against data from other regulatory agencies to assess the consistency of the observed changes in approval lag before and after the reforms. Records were standardized and matched using generic drug names, active ingredients, approval dates, and initial indications, with potential duplicates manually checked. Predefined operational definitions and classification rules were consistently applied to all records included in the analyses. Any discrepancies across sources or between the two authors were resolved through discussion based on official source documents. Information that could not be verified was recorded as missing and excluded only from the relevant analysis, with analysis-specific sample sizes reported.

### 2.4 Statistical analysis

For the purposes of comparative analyses, the period classification was anchored to the NMPA’s accession to the ICH in 2017 (Liu Y. et al., 2022). This event signified an institutional shift towards international regulatory harmonization, subsequently operationalized through a series of regulatory reforms (China Food and Drug Administration, 2017; National Medical Products Administration, 2024; Center for Drug Evaluation and National Medical Products Administration, 2018). Consequently, approvals granted between 2010 and 2017 were categorized as pre-reform, whereas those granted between 2018 and 2025 were categorized as post-reform. The year 2018 marked the first complete year following this regulatory transition and coincided with the establishment of the NHSA, a concurrent institutional milestone in reimbursement reform (Liu G. G. et al., 2022; Macabeo et al., 2023).

Continuous variables were summarized as medians and interquartile ranges, while categorical variables were presented as frequencies and percentages. The chi-square test or Fisher’s exact test was used to compare categorical variables between groups. The Mann–Whitney U test was used to compare review time and approval lag between the two periods. Kaplan-Meier analysis and the log-rank test were applied to assess differences in time to reimbursement between pre- and post-reform periods. Approval lag across the 4 R&D strategies was assessed using the Kruskal–Wallis H test, followed by pairwise comparisons with Bonferroni adjustment. The factors associated with inclusion in the NRDL were analyzed utilizing multivariable binary logistic regression. Ten drug-related and regulatory characteristics were selected as candidate predictors due to their potential relevance to NRDL inclusion. Multicollinearity was evaluated through variance inflation factors (VIFs), with values below five deemed acceptable. Model calibration was assessed via the Hosmer–Lemeshow test, while model discrimination was evaluated using the C-statistic along with its 95% confidence interval. The adequacy of the model was determined using the events-per-variable (EPV) ratio, with an EPV of at least 10 considered sufficient. Sensitivity analyses were conducted to evaluate the robustness of the findings, employing an alternative period-specific definition of NRDL inclusion and utilizing approval dates from the European Medicines Agency (EMA) (European Medicines Agency, 2026) and the Japan Pharmaceuticals and Medical Devices Agency (PMDA) (Pharmaceuticals and Medical Devices Agency, 2026) as alternative comparators for approval lag analyses. All statistical analyses were executed using IBM SPSS Statistics version 27.0, Origin 2021, and R version 4.4.1. A two-tailed P-value of less than 0.05 was considered to indicate statistical significance.

## 3 Results

### 3.1 Sample overview

Between 2010 and 2025, a total of 361 overseas new drugs were approved by the NMPA, of which 213 had been included in the NRDL (Supplementary Figure S2). Annual approval counts rose markedly after 2017 (Figure 1), accompanied by a steeper increase in the cumulative number of NRDL-listed drugs (Supplementary Figure S2). As shown in Table 1, the leading ATC classifications were antineoplastic and immunomodulating agents (147, 40.7%), followed by anti-infectives for systemic use (45, 12.5%) and alimentary tract and metabolism (37, 10.2%). Compared with the earlier period, approval counts increased in the later period, with the highest proportion observed across most ATC classes (Supplementary Figure S3). In terms of origin, overseas new drugs mainly originated from the United States (85, 23.5%), Germany (70, 19.4%) and Japan (37, 10.2%). Meanwhile the number of source countries increased from 14 to 22 between the two study periods (Supplementary Figure S4). For drug indications, lymphoma was the most common indication among the ten leading indications. Comparative analysis between the two periods revealed an evident shift toward oncology-related indications (Supplementary Figure S5). The approved overseas new drugs were predominantly formulated as injections (154, 42.7%) and tablets (138, 38.2%).

**FIGURE 1:**
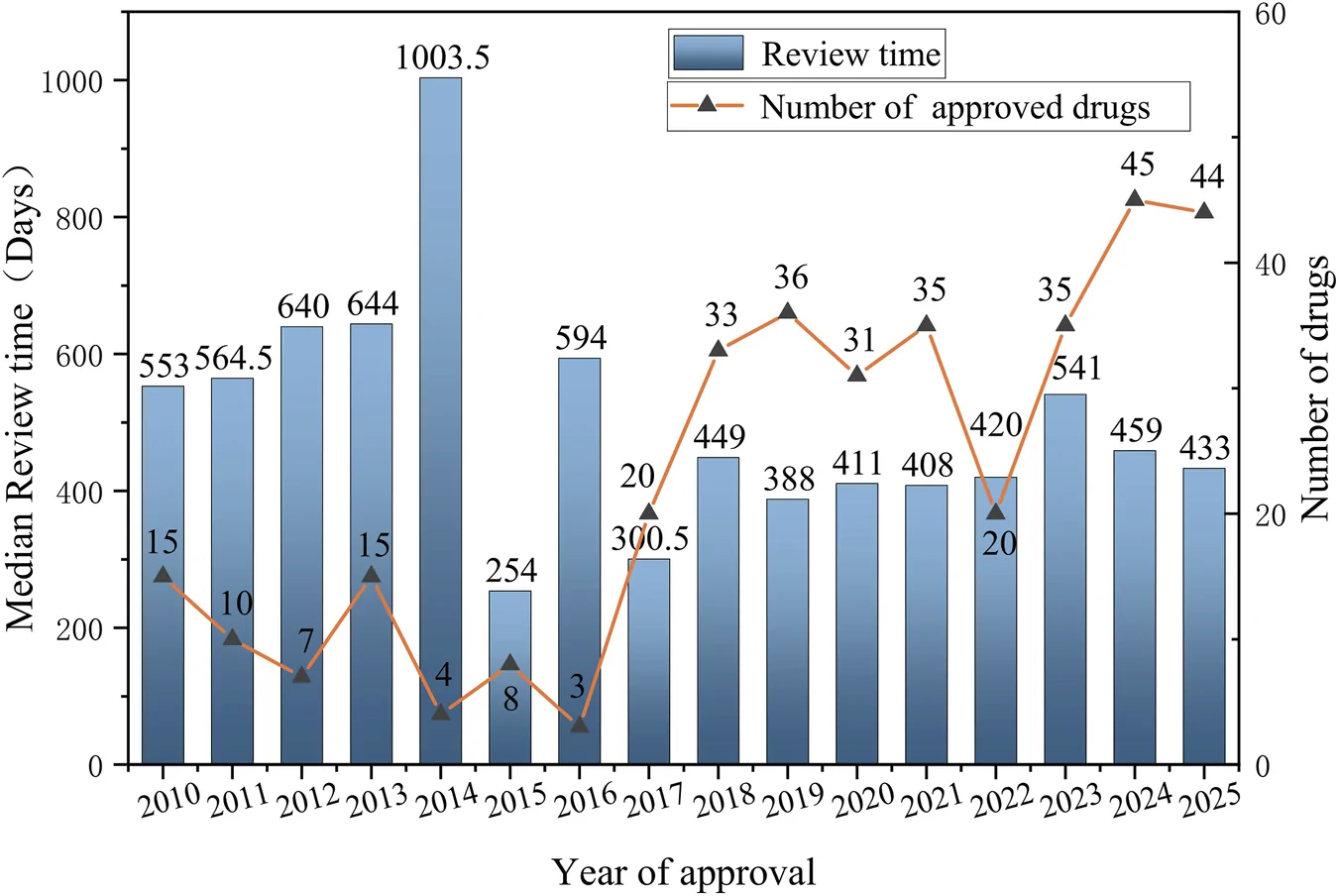
Annual review time and number of approved drugs from 2010 to 2025. Bars represent the annual median review time; the line represents the number of drugs approved in each year.

**TABLE 1:** Overview of NMPA-approved overseas new drugs.

Characteristic	N = 361 (%); n (%)
ATC classification	
Antineoplastic and immunomodulating agents	147 (40.7)
Anti-infectives for systemic use	45 (12.5)
Alimentary tract and metabolism	37 (10.2)
Nervous system	36 (10.0)
Blood and blood-forming organs	24 (6.6)
Cardiovascular system	18 (5.0)
Respiratory system	12 (3.3)
Systemic hormonal preparations	10 (2.8)
Musculo-skeletal system	9 (2.5)
Dermatologicals	6 (1.7)
Sensory organs	6 (1.7)
Various	6 (1.7)
Genitourinary system and sex hormones	5 (1.4)
Origin	
United States	85 (23.5)
Germany	70 (19.4)
Japan	37 (10.2)
Italy	31 (8.6)
Switzerland	26 (7.2)
Ireland	25 (6.9)
United Kingdom	21 (5.8)
Canada	18 (5.0)
France	13 (3.6)
Denmark	6 (1.7)
Sweden	6 (1.7)
Spain	5 (1.4)
Belgium	3 (0.8)
India	3 (0.8)
South Korea	3 (0.8)
Netherlands	2 (0.6)
Singapore	2 (0.6)
Austria	1 (0.3)
Finland	1 (0.3)
Croatia	1 (0.3)
Portugal	1 (0.3)
Slovenia	1 (0.3)
Dosage form	
Injection	154 (42.7)
Tablet	138 (38.2)
Capsule	47 (13.0)
Other	22 (6.1)
Indication (top 10)	
Lymphoma	17 (4.7)
Non-small cell lung cancer	15 (4.2)
Breast cancer	15 (4.2)
Type 2 diabetes	13 (3.6)
Hepatitis C	11 (3.0)
Psoriasis	9 (2.5)
Multiple sclerosis	9 (2.5)
Multiple myeloma	9 (2.5)
Leukemia	8 (2.2)
Rheumatoid arthritis	8 (2.2)

### 3.2 Characteristics of overseas new drugs

Between 2010 and 2025, chemical drugs constituted the majority of approvals (69.3%), but the proportion of biologics increased from 13.4% in the pre-reform period to 35.8% in the post-reform period (P < 0.001). In line with increasing attention to rare disease and pediatric drugs, the proportion of rare disease drugs increased significantly from 9.8% to 28.7% (P < 0.001). In contrast, pediatric-specific drugs remained scarce, although the proportion of drugs approved for pediatric use increased in the post-reform period. Following regulatory reforms, approvals of overseas innovative drugs and UNODs emerged after 2018. Notably, a marked increase was observed in oncology drugs, whose proportion in the pre-reform period (15, 18.3%) was significantly lower than in the post-reform period (84, 30.1%; P < 0.05). In terms of NRDL inclusion status, the proportion of included drugs was 67.1% in the pre-reform period and 56.6% in the post-reform period, with no statistically significant difference between the two periods (P = 0.091). Of the 361 approved overseas new drugs, 178 (49.3%) received at least one expedited-program designation. Compared with the pre-reform period (19.5%), the proportion of approved drugs receiving expedited program designations increased significantly in the post-reform period (58.1%). The range of expedited programs broadened after the reform (P < 0.001), with concurrent Priority Review (PR) and Conditional Approval (CA) representing the most common combination (13.6%; Table 2). These trends indicated that post-reform expansion extended beyond approval volume, encompassing shifts in the composition of approved drugs and broader adoption of expedited programs.

**TABLE 2:** The characteristics of approved overseas new drugs, 2010–2025.

Characteristics	N (%)			
	All (n = 361)	2010–2017 (n = 82)	2018–2025 (n = 279)	P value
Drug type	​	​	​	P < 0.001
Chemicals	250 (69.3)	71 (86.6)	179 (64.2)	​
Biologicals	111 (30.7)	11 (13.4)	100 (35.8)	​
Rare disease	​	​	​	P < 0.001
Yes	88 (24.4)	8 (9.8)	80 (28.7)	​
No	273 (75.6)	74 (90.2)	199 (71.3)	​
Innovativeness	​	​	​	P = 0.001
Innovative drug	33 (9.1)	0 (0.0)	33 (11.8)	​
Original drug	328 (90.9)	82 (100.0)	246 (88.2)	​
Indication population	​	​	​	P = 0.114
Children only	3 (0.8)	0 (0.0)	3 (1.1)	​
Adults only	289 (80.1)	72 (87.8)	217 (77.8)	​
Adults and children	69 (19.1)	10 (12.2)	59 (21.1)	​
UNOD^a^	​	​	​	P < 0.001
Yes	44 (12.2)	0 (0.0)	44 (15.8)	​
No	317 (87.8)	82 (100.0)	235 (84.2)	​
NRDL inclusion status^b^	​	​	​	P = 0.091
Yes	213 (59.0)	55 (67.1)	158 (56.6)	​
No	148 (41.0)	27 (32.9)	121 (43.4)	​
Therapeutic area	​	​	​	P = 0.035
Oncology	99 (27.4)	15 (18.3)	84 (30.1)	​
Non-oncology	262 (72.6)	67 (81.7)	195 (69.9)	​
Expedited program^c^	​	​	​	P < 0.001
Only PR	117 (32.4)	16 (19.5)	101 (36.2)	​
Only CA	5 (1.4)	0 (0.0)	5 (1.8)	​
Only BTD	1 (0.3)	0 (0.0)	1 (0.4)	​
PR and CA	38 (10.5)	0 (0.0)	38 (13.6)	​
PR and BTD	10 (2.8)	0 (0.0)	10 (3.6)	​
All	7 (1.9)	0 (0.0)	7 (2.5)	​
None	183 (50.7)	66 (80.5)	117 (41.9)	​

^a^ UNOD, refers to an overseas new drug included in any of the lists issued by the National Medical Products Administration.

^b^ NRDL, inclusion status was assessed as of 1 January 2026.

^c^ Expedited program includes Priority Review (PR), Conditional Approval (CA), and Breakthrough Therapy Designation (BTD).

### 3.3 Review time, approval lag status, and R&D strategies for overseas new drugs in China

To evaluate whether the post-reform alterations in the composition and regulatory pathways of approved drugs were associated with enhanced regulatory performance, a comparative analysis of review time and approval lag was conducted between the two periods. Post-reform, the median review time decreased significantly from 532 days during 2010–2017 to 427 days in 2018–2025 (P < 0.05) (Figure 2a). An annual trend analysis further revealed a pronounced reduction in review time for new drugs from overseas (Figure 1), particularly after 2017. Of the 361 overseas new drugs, 316 had corresponding FDA approval records and were included in the FDA-referenced approval-lag analysis, comprising 65 drugs approved between 2010 and 2017 and 251 approved between 2018 and 2025. The median approval lag significantly decreased from 1,442 days in the earlier period to 1,188 days in the later period (P < 0.05) (Figure 2b). Despite this reduction, the median approval lag remained considerable. Furthermore, sensitivity analyses using EMA and PMDA approvals as alternative comparators indicated that the median approval lag decreased from 1,304–986 days (P < 0.05) and from 1,025–774 days (P > 0.05), respectively (Supplementary Table S4).

**FIGURE 2:**
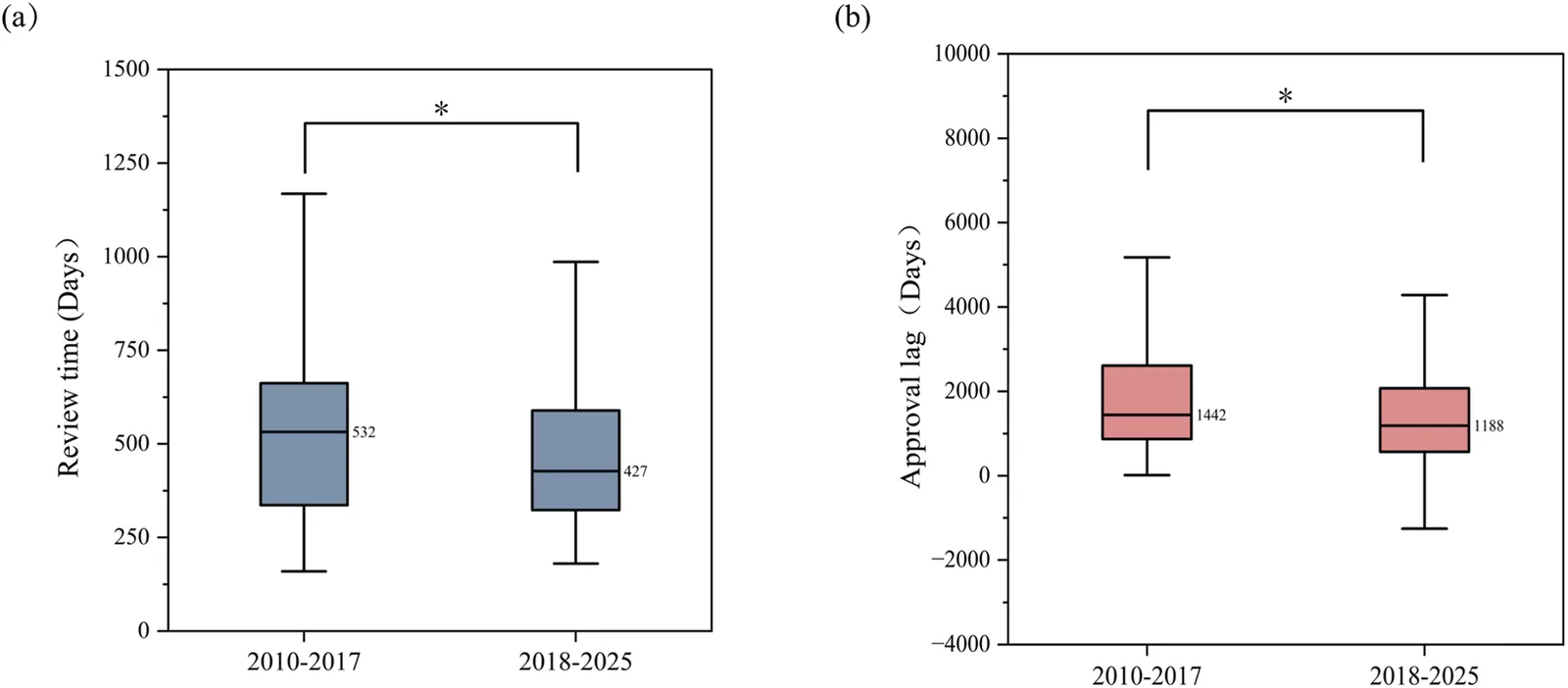
Review time and approval lag of approved overseas new drugs, 2010–2025. **(a)** Review time comparison between the two time periods. **(b)** Approval lag comparison between the two time periods. *P < 0.05; **P < 0.001.

To establish a connection between clinical development and regulatory access, we conducted an analysis of the R&D strategies employed for 279 drugs approved by the NMPA between 2018 and 2025. These strategies encompassed various methodologies for utilizing clinical evidence generated both internationally and within China to facilitate marketing approval. Subsequently, we compared the extent of approval lag across the four identified strategy groups. The comprehensive decision framework utilized to categorize drugs into the 4 R&D strategies was illustrated in Figure 3. Strategies A and B emerged as the most frequently employed, collectively constituting the majority of approvals annually (refer to Supplementary Figure S6). Drugs for rare diseases (57.4%) and UNODs (41.5%) represented a significantly larger proportion within Strategy A (P < 0.001). Furthermore, overseas innovative drugs comprised 26.2% of those adhering to Strategy B, a proportion significantly higher than that observed in the other three strategies (P < 0.001). Notably, variations were evident across therapeutic areas, with oncology drugs representing 73.9% of those following Strategy C (P < 0.001). Additionally, more than half of the drugs categorized under Strategies A, B, and C were recipients of at least one expedited program, in contrast to only 33.9% under Strategy D (see Table 3).

**FIGURE 3:**
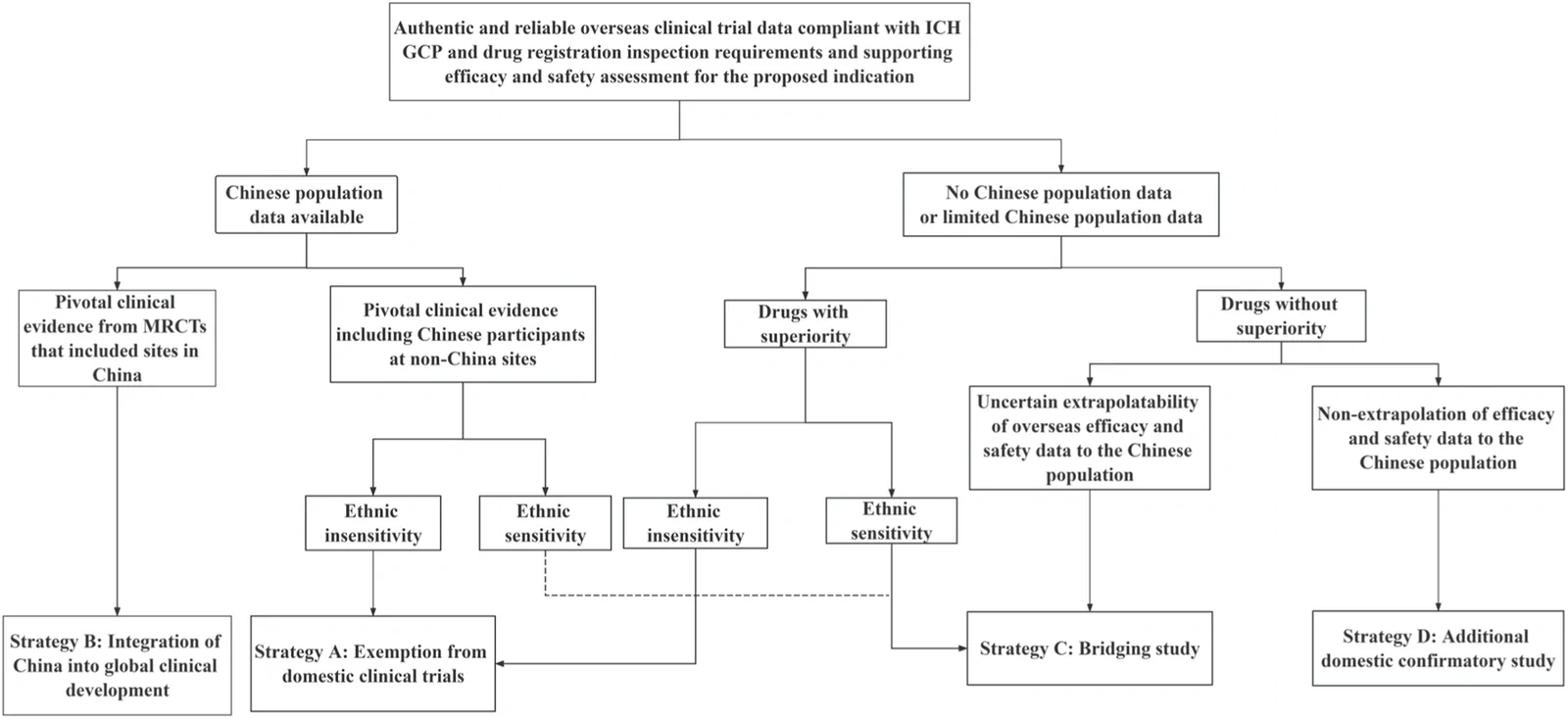
Decision framework for selecting R&D strategies for overseas new drugs in China. The framework was developed based on ICH E5 and E17 and relevant Chinese technical guidance to provide a regulatory reference for strategy selection. Dashed arrows indicate scenarios in which ethnic insensitivity has not been verified and a bridging study may therefore be required. Assessments of ethnic insensitivity and superiority were based on regulatory conclusions documented in CDE review reports. Drugs with evidence of lack of efficacy or serious safety concerns were not included in the strategy classification, as they are not recommended for clinical trials in China.

**TABLE 3:** Characteristics of overseas new drugs by R&D strategy, 2018–2025.

Characteristics	N (%)					
	All (n = 279)	Strategy A (n = 94)	Strategy B (n = 103)	Strategy C (n = 23)	Strategy D (n = 59)	P value
Drug type	​	​	​	​	​	P = 0.001
Chemicals	179 (64.2)	50 (53.2)	73 (70.9)	10 (43.5)	46 (78.0)	​
Biologicals	100 (35.8)	44 (46.8)	30 (29.1)	13 (56.5)	13 (22.0)	​
Rare disease	​	​	​	​	​	P < 0.001
Yes	80 (28.7)	54 (57.4)	15 (14.6)	2 (8.7)	9 (15.3)	​
No	199 (71.3)	40 (42.6)	88 (85.4)	21 (91.3)	50 (84.7)	​
Innovativeness	​	​	​	​	​	P < 0.001
Innovative drug	33 (11.8)	0 (0.0)	27 (26.2)	2 (8.7)	4 (6.8)	​
Original drug	246 (88.2)	94 (100.0)	76 (73.8)	21 (91.3)	55 (93.2)	​
UNOD^a^	​	​	​	​	​	P < 0.001
Yes	44 (15.8)	39 (41.5)	4 (3.9)	1 (4.3)	0 (0.0)	​
No	235 (84.2)	55 (58.5)	99 (96.1)	22 (95.7)	59 (100.0)	​
Therapeutic area	​	​	​	​	​	P < 0.001
Oncology	84 (30.1)	11 (11.7)	37 (35.9)	17 (73.9)	19 (32.2)	​
Non-oncology	195 (69.9)	83 (88.3)	66 (64.1)	6 (26.1)	40 (67.8)	​
Number of expedited programs^b^	​	​	​	​	​	P < 0.001
0	117 (41.9)	23 (24.5)	45 (43.7)	10 (43.5)	39 (66.1)	​
≥1	162 (58.1)	71 (75.5)	58 (56.3)	13 (56.5)	20 (33.9)	​

^a^ UNOD, refers to an overseas new drug included in any of the lists issued by the National Medical Products Administration.

^b^ Expedited program includes Priority Review (PR), Conditional Approval (CA), and Breakthrough Therapy Designation (BTD).

Among the 279 drugs categorized by R&D strategy, 251 had an associated FDA approval date and were thus included in the strategy-specific approval lag analysis. The median approval lag exhibited significant variation across the four strategies (P < 0.001; Supplementary Figure S7). Bonferroni-adjusted comparisons indicated that the approval lag for Strategy D was significantly longer than for Strategies A and C (P < 0.05) and B (P < 0.001). Additionally, Strategy A demonstrated a longer approval lag compared with Strategy B (P < 0.05). Upon examination of approval timing relative to the United States (refer to Supplementary Table S5), Strategy B exhibited the lowest proportion of drugs approved more than 3 years subsequent to US approval, at 40.8%. Notably, 5.1% of these drugs received approval in China prior to US approval, and 27.6% were approved within 1 year thereafter. The reduced approval lag associated with Strategy B underscored the significance of integrating China into global clinical development processes. In contrast, Strategy D demonstrated the highest proportion of drugs approved more than 3 years after US approval, at 79.2%, although it is noteworthy that one drug under Strategy D received earlier approval in China. For Strategies A and C, all drugs were approved in China later than in the United States, with 97.6% and 86.4% of the drugs, respectively, being approved more than 1 year later.

### 3.4 Reimbursement access for overseas new drugs in China

During the study period, eight official versions of the NRDL were released in China. A Kaplan-Meier analysis of all 361 drugs revealed that the median time to reimbursement was significantly shorter in the period from 2018 to 2025 compared to 2010 to 2017 (743 days *versus* 2,297 days; P < 0.001; see Supplementary Figure S8). To further investigate the factors influencing the inclusion of overseas new drugs in the NRDL, a multivariable binary logistic regression analysis was performed. This model encompassed 213 NRDL inclusion events and 10 predictor variables, resulting in an EPV ratio of 21.3, thereby affirming the model’s adequacy. The Hosmer–Lemeshow test indicated no significant lack of fit (χ^2^ = 11.504, df = 8, P = 0.175), and the C-statistic of 0.702 (95% CI: 0.649–0.756) demonstrated acceptable discriminative ability. All VIF values were below 1.5, suggesting an absence of substantial multicollinearity among the predictors. The final model (refer to Table 4) identified that drug class, therapeutic area, UNOD status, indication population, and CA designation were significantly associated with NRDL inclusion. Specifically, it was determined that biological drugs had lower odds of being included in the NRDL compared to chemical drugs (OR = 0.464, P = 0.003), whereas drugs with oncology status were more likely to be included (OR = 2.125, P = 0.016).

**TABLE 4:** Binary logistic regression analysis of factors associated with NRDL inclusion.

Variable	N	%	OR	95% CI	P value
Drug class					
Chemicals	250	69.3	References	​	​
Biologicals	111	30.7	0.464	0.281–0.767	P = 0.003
Approval year^a^					
Pre-reform period	82	22.7	References	​	​
Post-reform period	279	77.3	0.699	0.388–1.262	P = 0.235
Therapeutic area					
Non-oncology	262	72.6	References	​	​
Oncology	99	27.4	2.125	1.149–3.928	P = 0.016
Innovativeness					
Original drug	328	90.9	References	​	​
Innovative drug	33	9.1	0.928	0.397–2.172	P = 0.864
Rare disease					
No	273	75.6	References	​	​
Yes	88	24.4	0.796	0.442–1.435	P = 0.448
UNOD^b^					
No	317	87.8	References	​	​
Yes	44	12.2	2.924	1.302–6.566	P = 0.009
Target population					
Adults only	289	80.1	References	​	​
Pediatric patients included^c^	72	19.9	0.464	0.254–0.847	P = 0.012
PR^d^					
No	189	52.3	References	​	​
Yes	172	47.7	1.518	0.886–2.602	P = 0.129
CA^d^					
No	311	86.1	References	​	​
Yes	50	13.9	0.327	0.152–0.705	P = 0.004
BTD^d^					
No	343	95.0	References	​	​
Yes	18	5.0	0.846	0.273–2.625	P = 0.772

Outcome was NRDL, inclusion status as of 1 January 2026.

^a^ Approval year refers to the year of initial approval by the NMPA.

^b^ UNOD, refers to an overseas new drug included in any of the lists issued by the National Medical Products Administration.

^c^ Pediatric patients included refers to drugs indicated either exclusively for pediatric patients or for both adult and pediatric patients.

^d^ PR, priority review; CA, conditional approval; BTD, breakthrough therapy designation.

Furthermore, the status of UNOD was identified as a significant determinant. Drugs classified as UNODs were more likely to be included compared to those not identified as such (OR = 2.924, P = 0.009). Regarding the target population, drugs indicated for pediatric patients demonstrated lower odds of inclusion in the NRDL compared to those indicated solely for adults (OR = 0.464, P = 0.012). Within expedited programs, drugs with a CA designation exhibited lower odds of NRDL inclusion than those without such a designation (OR = 0.327, P = 0.004). The sensitivity analysis corroborated the robustness of our primary findings, with the exception of oncology status and approval period (Supplementary Table S6). Collectively, these results indicated that reimbursement access had accelerated in the post-reform period, although NRDL inclusion continued to vary based on product characteristics and regulatory designations.

## 4 Discussion

### 4.1 The evolving landscape of overseas new drug approvals

Our analysis showed that annual approval counts for overseas new drugs remained low until 2017. The unusually high median review time observed in 2014 may reflect the concentration of long-pending applications within a small annual approval cohort. In 2015, the Chinese regulatory authority issued the “Announcement on Conducting Self-Inspection and Verification of Drug Clinical Trial Data” to address non-compliance in clinical trial data (China Food and Drug Administration, 2015), with approvals further declining to only three in 2016. Following the implementation of regulatory reforms, annual approval counts rose markedly after 2017, while regulatory review times shortened. Despite this expansion, overseas innovative drugs accounted for only 9.1% of approvals (Table 2), suggesting that the post-reform expansion may have partly reflected the clearance of a pre-existing application backlog (Xu et al., 2018).

Although chemical drugs remained predominant, the proportion of biologics increased markedly during the later period, consistent with the global shift toward biologic therapies and growing clinical demand in China (Tan et al., 2025). The therapeutic landscape evolved in parallel, with oncology and rare disease therapies becoming increasingly prominent. The growing prominence of oncology drugs may reflect the growing cancer burden in China, where cancer has remained one of the leading causes of death, thereby prompting greater attention to oncology areas (Qi et al., 2023). The increase in rare disease drug approvals may be attributable to multiple policies introduced in China and the NMPA’s designation of rare disease drugs as one of the pivotal areas encouraged for R&D (Zhao et al., 2023).

The evolving profile of approved drugs appeared to more closely align with the intended objectives of expedited pathways (Lan et al., 2025), with 58.1% of post-reform approvals qualifying for at least one such pathway. However, performance varied among the different pathway subgroups. Breakthrough Therapy Designation (BTD) drugs exhibited the shortest approval lag and the most rapid time to reimbursement. PR drugs achieved the highest inclusion rate in the NRDL, whereas CA drugs had the lowest inclusion rate and the longest time to reimbursement (Supplementary Table S7). Furthermore, the availability of pediatric-specific drugs remained limited. This disparity may be attributed to the intrinsic challenges associated with pediatric drug development, including high technical thresholds, extended research and development timelines, difficulties in conducting clinical trials within pediatric populations, and limited commercial returns (Singh et al., 2025).

### 4.2 Approval lag status and R&D strategies for overseas new drugs

In addition to the rise in approval volume and the reduction in review durations, the post-reform era was marked by a decrease in approval lag. There was a notable decline in FDA-referenced approval lag, and a comparable significant reduction was evident in the EMA-referenced sensitivity analysis. Although the median approval lag referenced by the PMDA also diminished, this reduction did not reach statistical significance. This outcome may partially indicate concurrent advancements in drug approval timelines within Japan, potentially constraining the relative changes observed between China and Japan (Xu et al., 2026; Luo et al., 2023a).

A significant aspect of the reforms involved an enhanced regulatory acceptance of international clinical evidence. The expansion in the number of source countries for approved drugs from 14 to 22 across the two periods confirmed this observation. Nonetheless, the relevance of such evidence varied among products, as evidenced by the employment of distinct R&D strategies for securing regulatory approval in China. Prior research has indicated that R&D strategy serves as a crucial analytical framework for comprehending approval lag (Zhu and Chen, 2024), as varying strategies reflect both the timing and extent of China’s involvement in global development initiatives and the level of China-specific evidence required for regulatory approval. In accordance with this, our findings demonstrated variations in drug characteristics and approval lag across the four strategies.

Exemption from domestic clinical trials was used predominantly for rare disease drugs and UNODs. This pattern suggested that regulatory flexibility was selectively directed toward drugs targeting urgent unmet clinical needs, rather than indicating a general relaxation of evidence requirements (Mao et al., 2025; Wei et al., 2025). Nevertheless, the median approval lag under this strategy was 1,069 days (Supplementary Figure S7), significantly longer than that observed when China was integrated into global clinical development, and 48.2% of these drugs received NMPA approval more than 3 years after FDA approval (Supplementary Table S5). Although this finding may appear inconsistent with the intended role of the pathway in accelerating access, it likely reflected the fact that many of these drugs had already been marketed overseas for several years. Notably, none of the overseas innovative drugs received an exemption from domestic clinical trials. Thus, this pathway appeared to have functioned primarily as a retrospective catch-up mechanism (Wei et al., 2025) rather than as a route to synchronized global development.

In contrast, China’s integration into global clinical development via MRCTs constituted a more forward-looking and efficient pathway, facilitated by regulatory transitions. This pathway encompassed the highest proportion of innovative drugs from overseas and exhibited the shortest median approval lag, recorded at 737.5 days (refer to Supplementary Figure S7). Furthermore, drugs developed through this pathway demonstrated the highest proportion of approvals either preceding or occurring within 1 year of U.S. approval (as detailed in Supplementary Table S5). These findings indicated that multinational sponsors were increasingly incorporating China into their global development strategies. However, the persisting median lag of approximately 2 years suggested that China may still be integrated relatively late in some global development programs (Zhang et al., 2022), thereby limiting the potential for MRCT participation to achieve near-synchronous submission and approval.

Bridging studies remained an important pathway, particularly for oncology drugs, for which potential ethnic variability in pharmacokinetics and pharmacodynamics may limit the direct extrapolation of overseas evidence (Kogure et al., 2017; Wang et al., 2022). Bridging studies had a median approval lag of 1,289.5 days (Supplementary Figure S7), which was significantly shorter than that associated with additional domestic clinical trials and not significantly different from Strategies A or B. These findings suggested that, when early participation in MRCTs or reliance solely on overseas evidence was not feasible, bridging studies might serve as a pragmatic intermediate pathway that facilitated earlier market entry. However, bridging studies were typically initiated after pivotal overseas trials had been completed (Han et al., 2024), which might account for the substantial approval lag.

Drugs necessitating supplementary domestic clinical trials exhibited the most unfavorable approval-lag profile, characterized by a median delay of 2,066 days (Supplementary Figure S7), which was significantly longer than the delays associated with other strategies. Furthermore, 79.2% of these drugs received approval from the NMPA more than 3 years subsequent to their approval by the FDA (Supplementary Table S5). The extended delay observed in Strategy D may partially be attributed to the additional time required for the generation of domestic clinical evidence. Additionally, historical regulatory requirements and protracted review timelines have constituted substantial barriers to market entry for multinational pharmaceutical companies. As a result, some drugs completed their development overseas or received approval based on limited clinical evidence from Chinese patients, potentially necessitating separate domestic pivotal trials to support their approval in China (Mao et al., 2025; Zhang et al., 2022).

### 4.3 Reimbursement access and its determinants for overseas new drugs

Our study evaluated the temporal changes in reimbursement duration throughout the study period, revealing a significantly reduced interval in the post-reform era. A previous investigation similarly noted a decrease in the median time from regulatory approval to inclusion in the NRDL, from 4.1 years for cancer drug indications approved between 2011 and 2016 to 1.1 years for those approved between 2017 and 2022 (Zhang et al., 2026). Aligning with this trend, our findings indicated that new drugs from overseas were incorporated into the NRDL more swiftly in the post-reform period, with a median interval of 743 days (approximately 2.0 years). Although direct comparisons across different contexts and timeframes should be approached with caution, our results implied that the reimbursement timeline for overseas new drugs in China remained longer than those reported in Japan and certain European countries. This discrepancy may partially be attributed to variations in reimbursement processes. In selected European countries, structured Health Technology Assessment (HTA) timelines, early-access pathways, and managed entry agreements facilitate prompt reimbursement. In Japan, the integration of statutory timelines for new drug reimbursement, coupled with early-stage technical guidance and concurrent coordination between regulatory review and reimbursement assessment, has facilitated expedited access to the national reimbursement system for pharmaceuticals (Wang et al., 2025; Takayama and Narukawa, 2016).

The enhancements observed following the reforms extended beyond regulatory review to encompass reimbursement access. Nevertheless, these improvements were more pronounced in terms of the expedited inclusion in the NRDL rather than the likelihood of inclusion. During the post-reform period, approvals exhibited a tendency towards reduced odds of NRDL inclusion in the primary cutoff-date analysis (OR = 0.699), although this association did not reach statistical significance (P = 0.235). This seemingly paradoxical pattern may be attributed to differential follow-up rather than an actual policy effect, as the fixed cutoff and annual NRDL adjustment cycle provided recently approved drugs with fewer opportunities for inclusion. Supporting this interpretation, post-reform approval was associated with increased odds of NRDL inclusion in the sensitivity analysis (OR = 2.923, P < 0.001; see Supplementary Table S6).

Drug class was consistently associated with NRDL inclusion across binary logistic regression analysis (Table 4) and sensitivity analysis (Supplementary Table S6), with chemical drugs showing higher odds of inclusion than biologicals. Although biologicals have been designated as a priority area for drug innovation, their inclusion in the NRDL may still be constrained by high treatment costs, substantial budget impact, and relatively limited market competition (Tan et al., 2025; Luo et al., 2023b). The oncology status demonstrated a correlation with increased likelihood of inclusion in the NRDL; however, this relationship was diminished and lost statistical significance in the sensitivity analysis (OR = 1.551, P = 0.134; Supplementary Table S6). This reduction in significance may partially be attributed to delays in the reimbursement process for oncology medications (Zhu and Chen, 2023). Despite advancements in national reimbursement negotiations enhancing access to anticancer drugs, the coverage remains selective, with reimbursement decisions being influenced by factors such as clinical benefits, acquisition costs, and the size of the target population (Wen et al., 2023; Zhang et al., 2021). Moreover, UNODs remained consistently associated with higher odds. This was consistent with the objective of China’s UNOD policy, which was introduced to further reduce the delay of overseas new drugs with urgent clinical needs and significant therapeutic benefits, thereby aligning closely with the core access criteria for NRDL inclusion (Zhu and Chen, 2025). Notably, drugs indicated exclusively for adults were also more likely to achieve NRDL inclusion than those with pediatric patients. This discrepancy may reflect the generally more mature and comprehensive clinical evidence base available for adult indications. In contrast, drugs for both adult and pediatric indications may face substantially higher barriers because of the limited availability of high-quality pediatric clinical evidence (van der Zanden et al., 2022), which makes it more difficult to satisfy the requirements for demonstrated clinical benefit and cost-effectiveness during reimbursement assessment. Evidentiary challenges may also contribute to the lack of reimbursement advantage observed for conditionally approved and rare disease drugs. Conditionally approved drugs were often authorized on the basis of surrogate endpoints or immature clinical data (Luo et al., 2023c), whereas clinical evidence supporting rare disease drugs was often sparse and subject to considerable uncertainty due to small patient populations and difficulties in conducting adequately powered trials (Qiao et al., 2022). These uncertainties regarding efficacy and safety may complicate reimbursement assessment and contribute to more cautious decision-making, potentially affecting reimbursement access for these drugs.

### 4.4 Implications for policy and practice

The observed disparities in approval lag across various R&D strategies underscored the critical need to transition from supplementing evidence at later stages to proactively planning China-specific clinical evidence, thereby facilitating earlier market entry for overseas new drugs. To effectuate this transition, it is imperative to prioritize the earlier inclusion of China in MRCTs through improved early dialogue between sponsors and regulators, streamlined administrative processes for human genetic resources, enhanced cross-departmental coordination, and the ongoing refinement and consistent application of the regulatory framework governing the evaluation of MRCT-generated evidence. Additionally, the regulatory flexibility currently afforded to overseas new drugs in China should be extended beyond the presently prioritized categories. In cases where bridging evidence remains necessary, efforts should focus on improving the predictability and efficiency of this pathway through the provision of clearer guidance, standardized assessment tools, and structured consultation mechanisms.

Despite the reduced time to reimbursement following recent reforms, neither conditionally approved drugs nor those for rare diseases demonstrated a significant advantage in terms of reimbursement. These findings indicated that merely accelerating procedural timelines may not sufficiently address the evidence-related barriers to coverage. At the reimbursement stage, policy focus should shift from one-time listing decisions to a lifecycle-based evidence framework. Drugs that meet significant unmet clinical needs but enter the reimbursement assessment with remaining evidentiary uncertainties may face challenges in obtaining timely reimbursement, potentially leaving some patients without affordable access to necessary therapies. Consequently, further efforts should concentrate on developing tailored reimbursement pathways. This can be accomplished by linking initial coverage to the ongoing generation of real-world evidence (RWE) and subsequent reassessment. Implementing such pathways would necessitate fit-for-purpose HTA guidance and standardized methodological and quality requirements for RWE, thereby enabling evidence on real-world effectiveness, safety, and resource utilization to inform both initial coverage and renewal decisions (Zhu and Chen, 2023; Wang et al., 2023).

In response to the affordability challenges associated with high-cost therapies, China has introduced its first Commercial Health Insurance Innovative Drug Catalog as a supplementary policy mechanism for innovative medicines not included in the NRDL (National Healthcare Security Administration & Ministry of Human Resources and Social Security, 2025). This catalog emphasizes innovative drugs that exhibit a high degree of innovation, substantial clinical value, and significant patient benefit, yet fall outside the coverage of basic medical insurance. Its purpose is to guide coverage by commercial health insurance and medical mutual-aid schemes, as the medicines listed are not reimbursed by the basic medical insurance fund. Future policy initiatives should focus on translating catalog inclusion into effective patient access through dynamic catalog adjustment, tiered benefit design, data-supported value assessment, streamlined settlement processes, and risk-sharing arrangements between insurers and pharmaceutical manufacturers.

## 5 Conclusion

China’s drug policy reforms were associated with improvements in access to overseas new drugs, as reflected by increased approvals, reduced approval lag, and expedited reimbursement processes. However, progress toward synchronized global development remained inconsistent. Variations in R&D strategies suggested that early involvement in MRCTs provided the most direct pathway to accelerated market entry, while delayed initiation of bridging studies or domestic evidence generation may continue to cause postponements. Although reimbursement processes for overseas new drugs have become more timely, NRDL inclusion remains contingent upon clinical evidence, therapeutic necessity, affordability, and budgetary impact. Achieving further advancements will necessitate the earlier integration of China into global development programs, the establishment of clearer and more predictable pathways for utilizing international evidence, and enhanced coordination between regulatory review and reimbursement decision-making. Such alignment is crucial to converting regulatory efficiency into equitable and sustainable patient access to overseas new drugs.

## 6 Limitations

This study was subject to several limitations. First, restricting the primary analysis to drugs with FDA approval records may have introduced selection bias and underestimated approval lag, while the lack of statistical significance in the PMDA-based sensitivity analysis raised uncertainty about the robustness of the estimated reduction. Second, potential subjectivity in R&D strategy classification and limited CDE review reports from 2010 to 2017 may have constrained the assessment of reform-related changes in R&D strategies. Third, unmeasured determinants may have reduced the model’s explanatory power, while unequal follow-up may have led to an underestimation of the probability of NRDL inclusion among recently approved drugs. Finally, the classification of regulatory changes into pre-reform and post-reform periods was relatively broad, and this approach may not fully capture the specific effects of individual regulatory reforms implemented at different time points.

## Data Availability

All relevant data presented in the study are included in the article/Supplementary Material, further inquiries can be directed to the corresponding author.
