# Supplementary material for: Regulatory reforms and the impact on overseas new drug accessibility in China: an analysis from 2010 to 2025
Source: Front Pharmacol. 2026 Oct 16;17:1961582. doi: 10.3389/fphar.2026.1961582 (PMC13720950; doi:10.3389/fphar.2026.1961582)
Supplement: Supplementary file 1 [file DataSheet1.pdf]

Supplementary Material

1 Supplementary Figures and Tables

1.1 Supplementary Figures

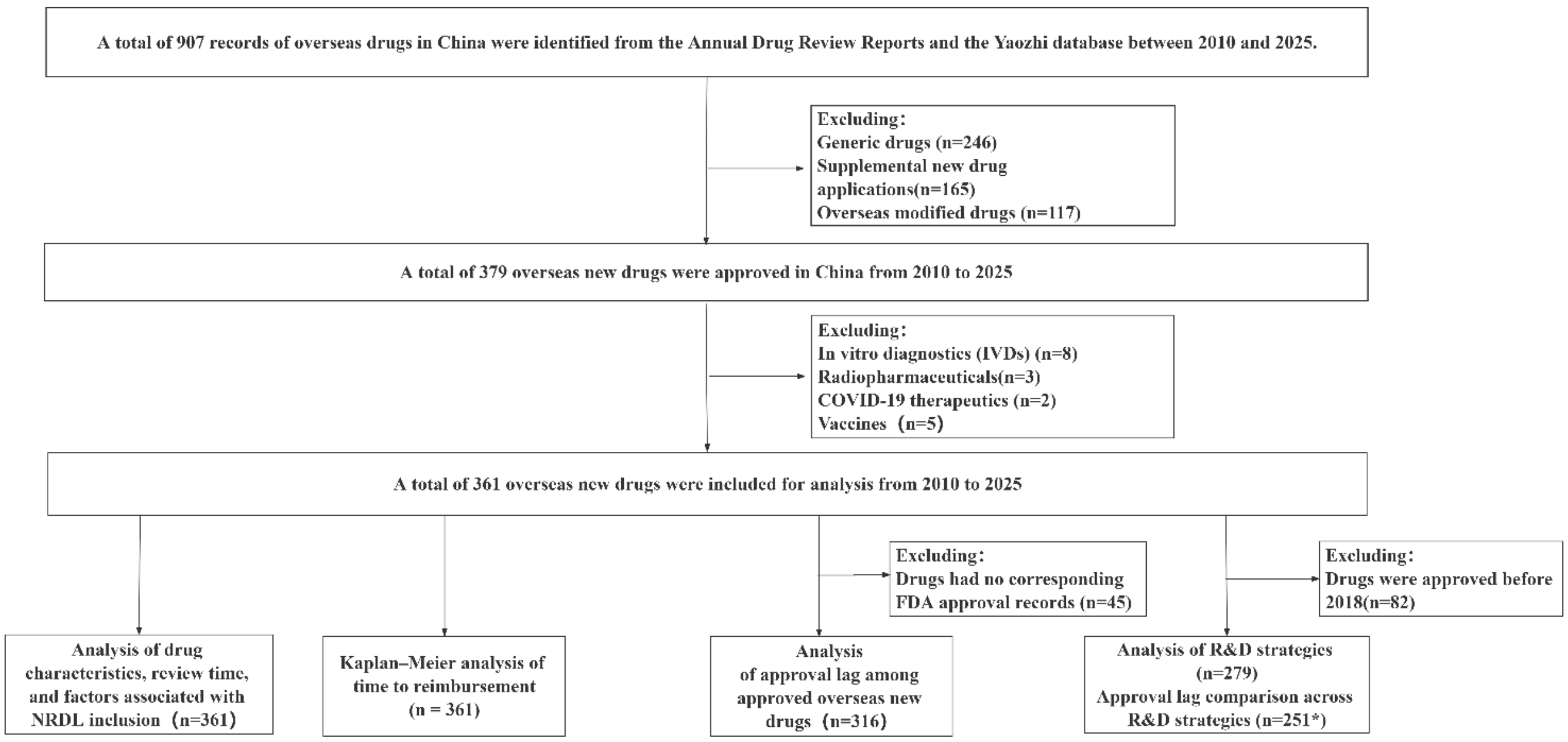

Note: *Among the 279 items, 251 had corresponding FDA approvals and complete approval-date data and were included in the strategy-specific approval lag analysis.

Supplementary Figure S1 Flow diagram of drug selection and analytical sample derivation.

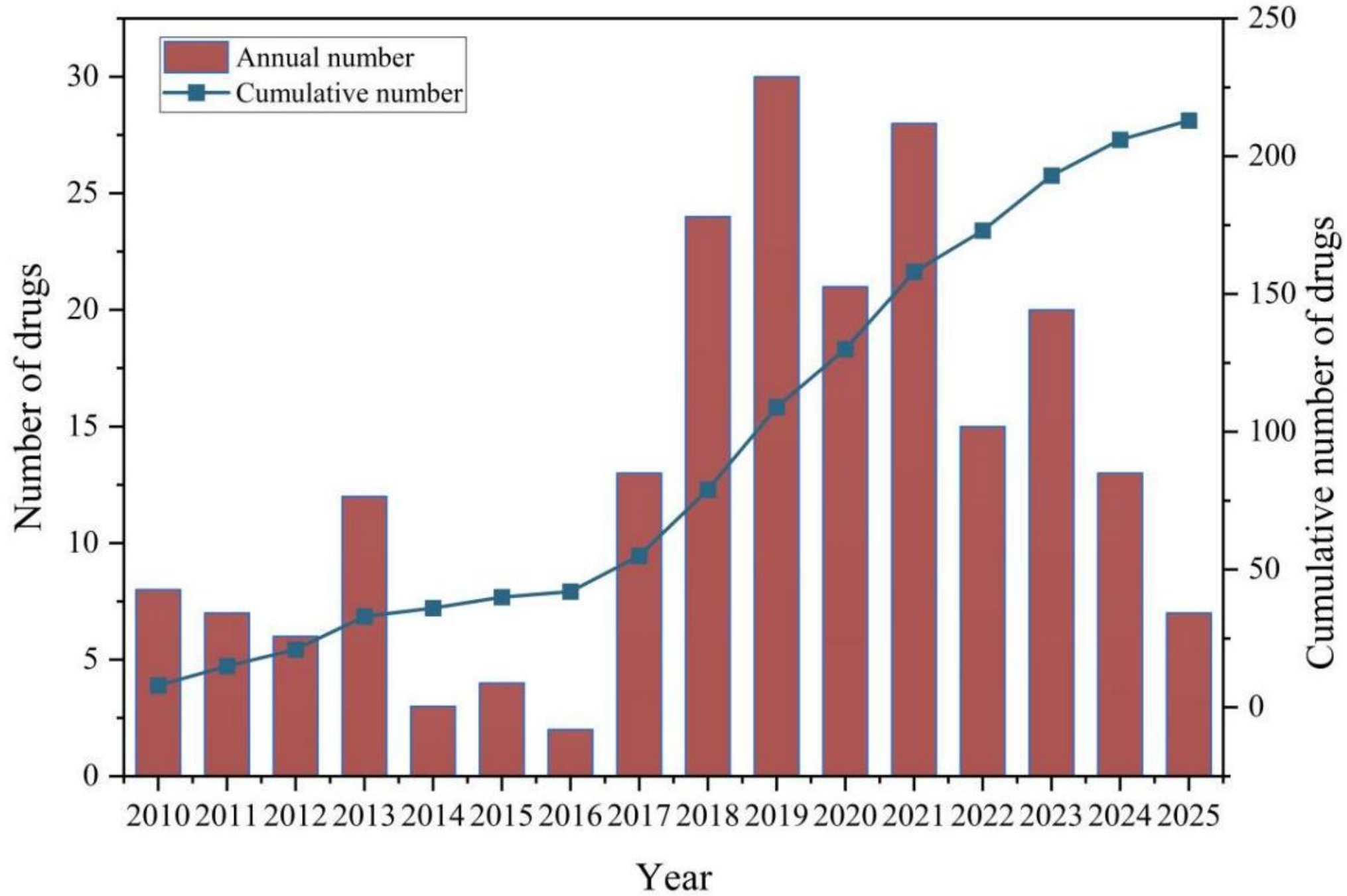

Supplementary Figure S2 Annual and cumulative numbers of overseas new drugs included in the NRDL by approval year, 2010–2025.

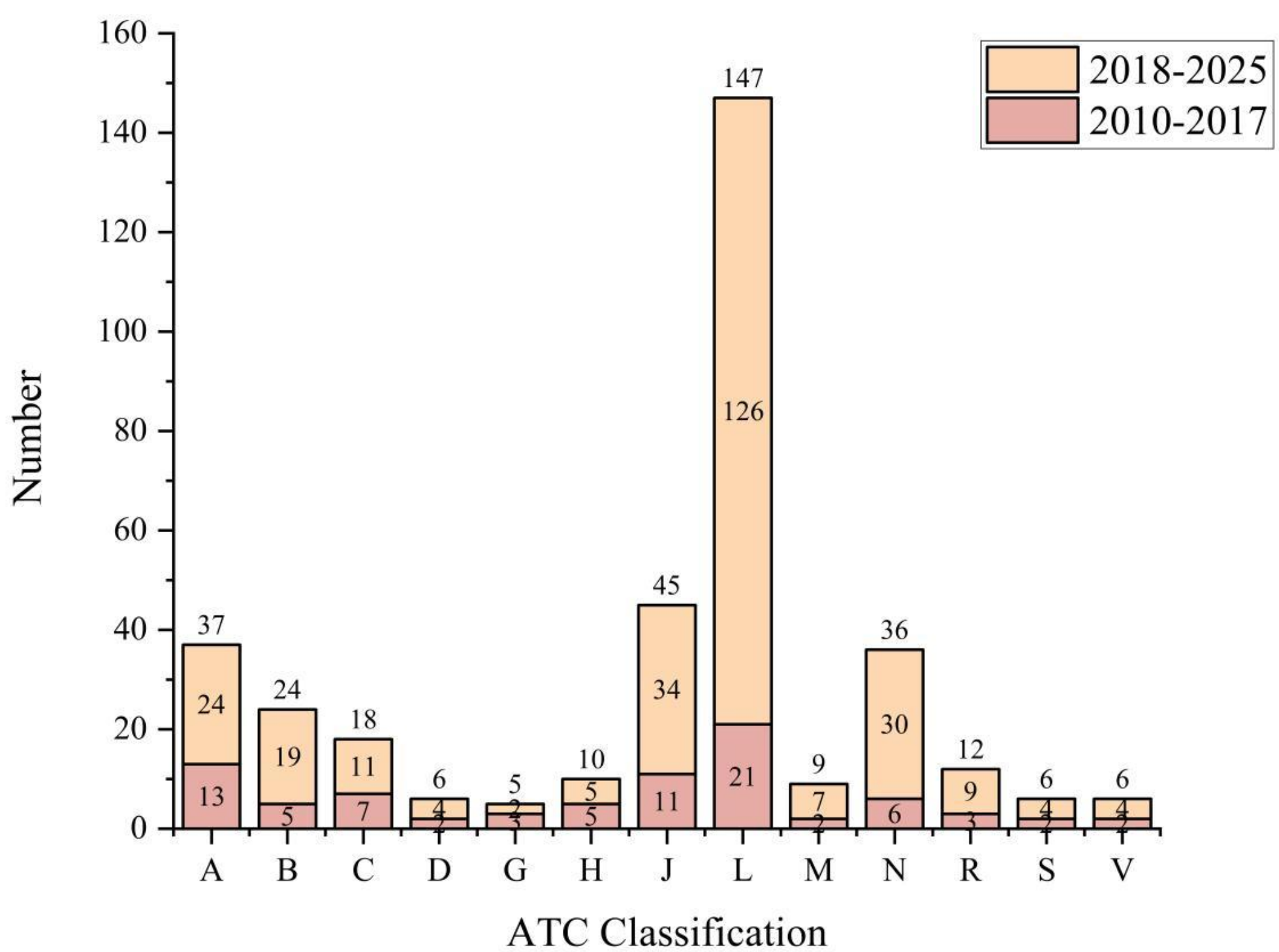

Note: A: Alimentary tract and metabolism; B: Blood and blood forming organs; C: Cardiovascular system; D: Dermatologicals; G: Genitourinary system and sex hormones; H: Systemic hormonal preparations, excluding sex hormones and insulins; J: Anti-infectives for systemic use; L: Antineoplastic and immunomodulating agents; M: Musculo-skeletal system; N: Nervous system; R: Respiratory system; S: Sensory organs; V: Various

Supplementary Figure S3 Distribution of ATC classifications by approval period, 2010–2017 and 2018–2025.

(a) (b)

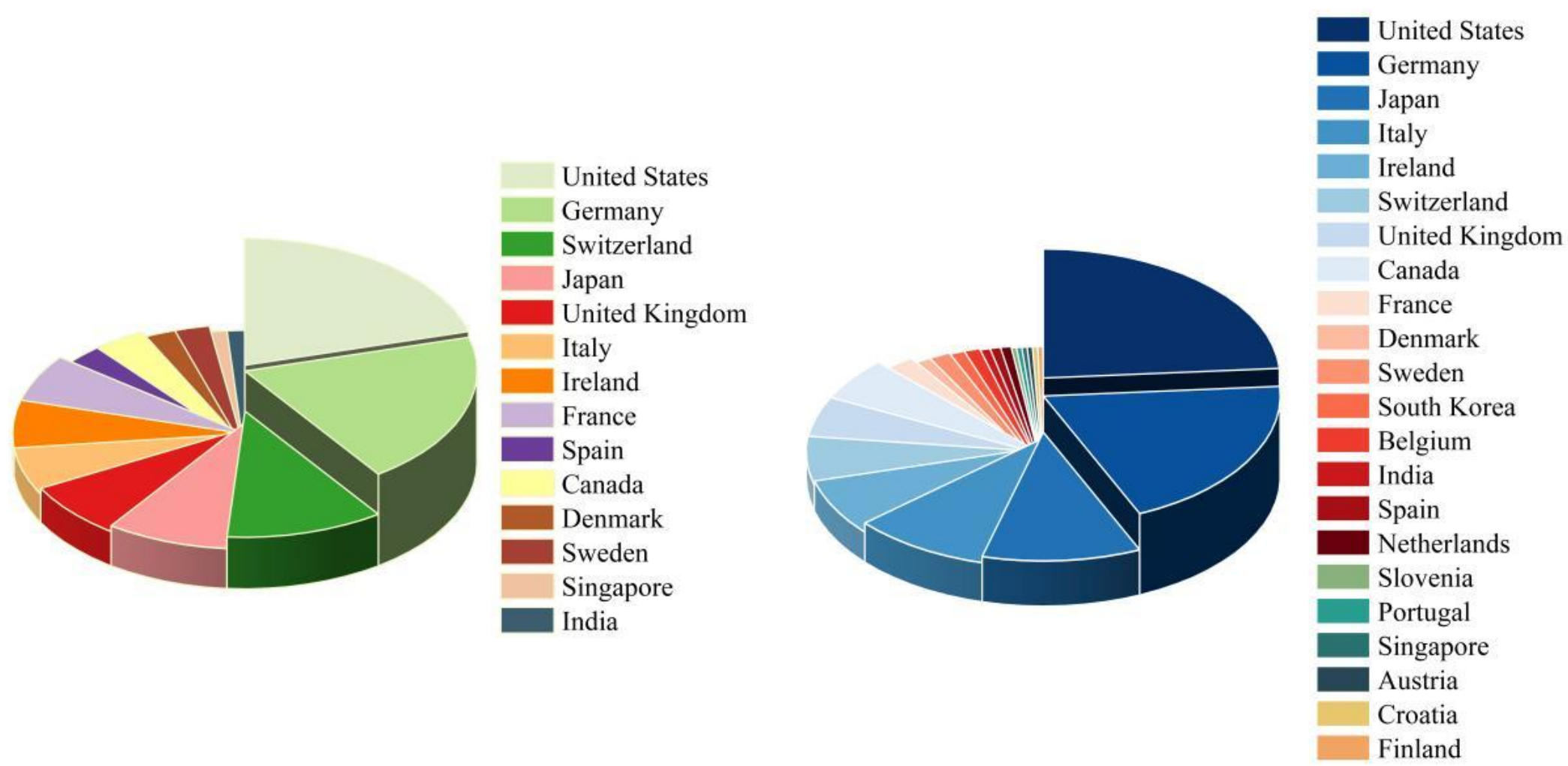

Supplementary Figure S4 Number of origin of overseas new drugs between the two periods. (a) Number of origin from 2010 to 2017. (b) Number of origin from 2018 to 2025.

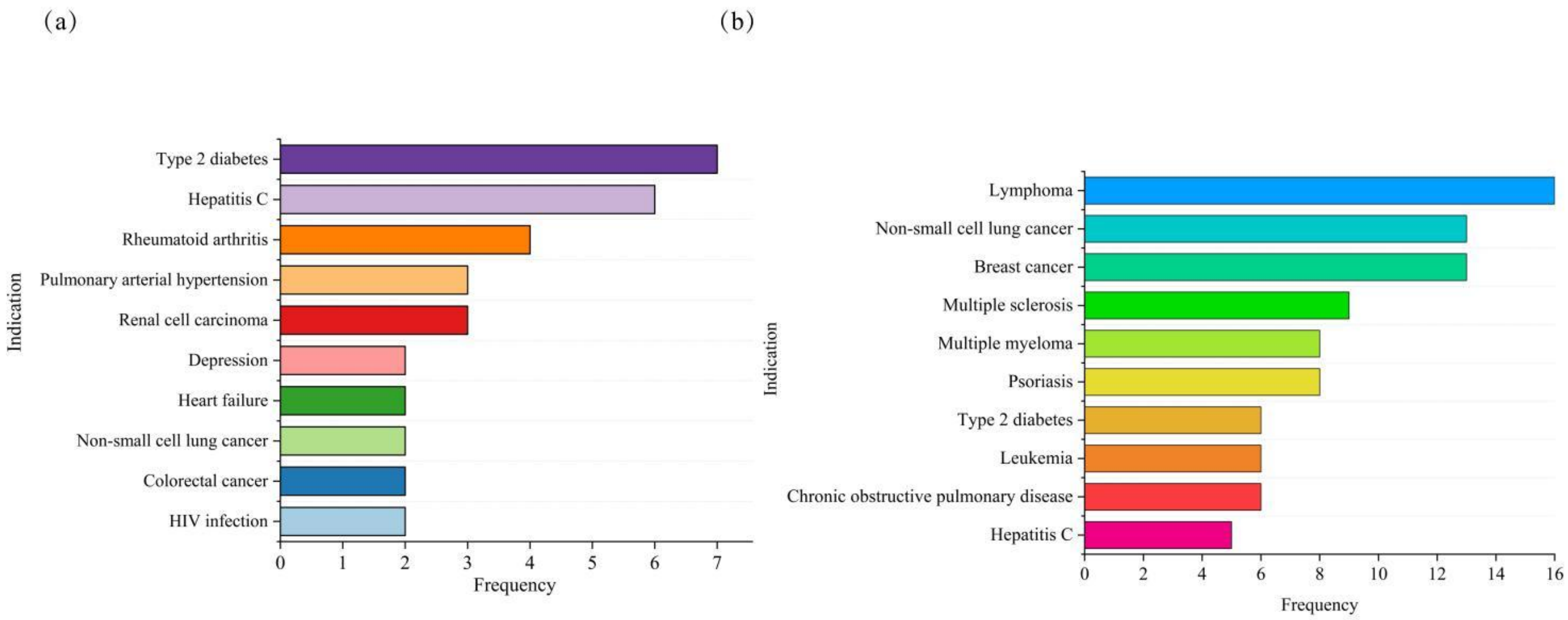

Supplementary Figure S5 Frequency of the top 10 indications between the two periods. (a) Frequency of the top 10 indications from 2010 to 2017. (b) Frequency of the top 10 indications from 2018 to 2025.

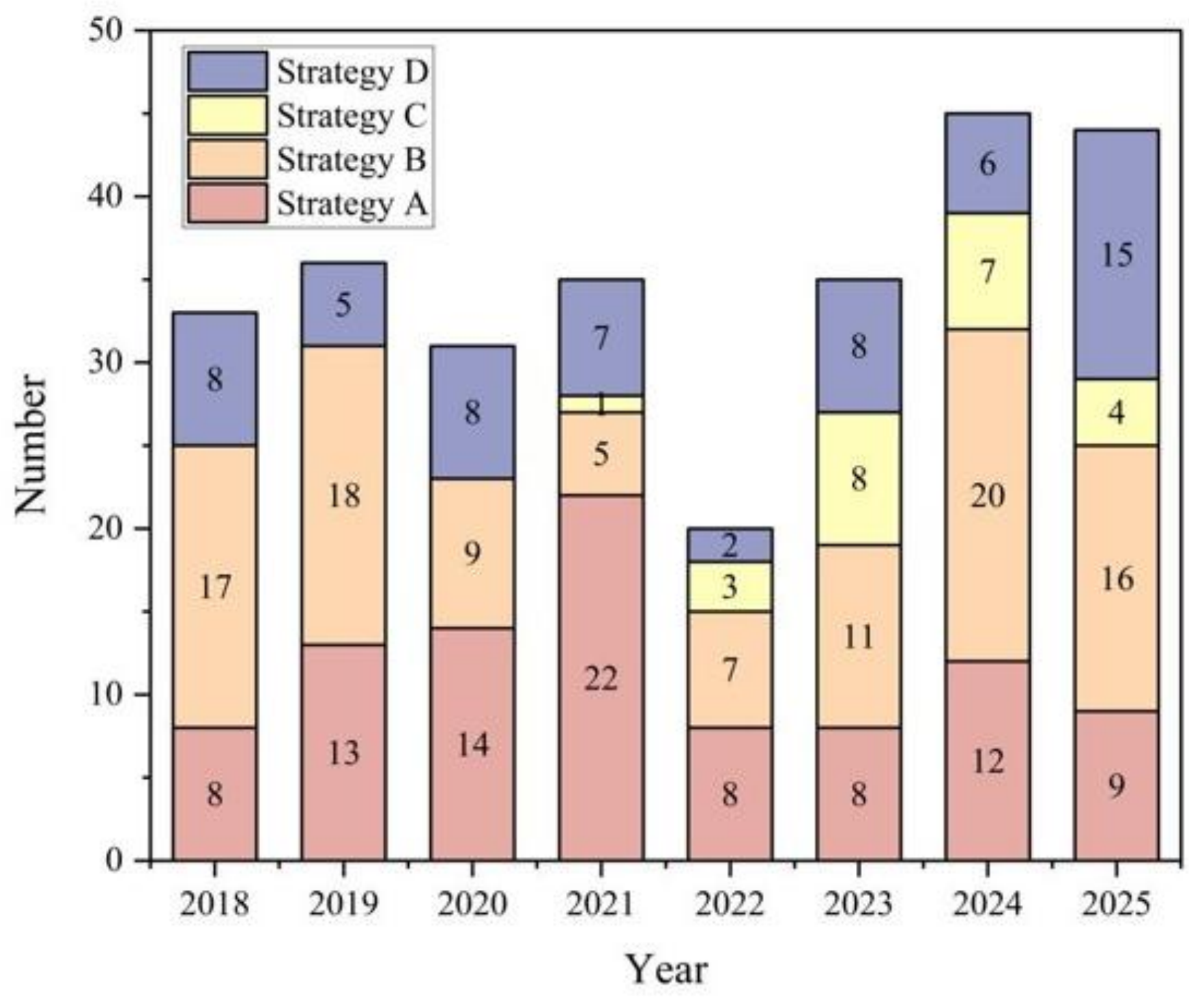

Supplementary Figure S6 Annual distribution of R&D strategies for overseas new drugs approved in China, 2018–2025.

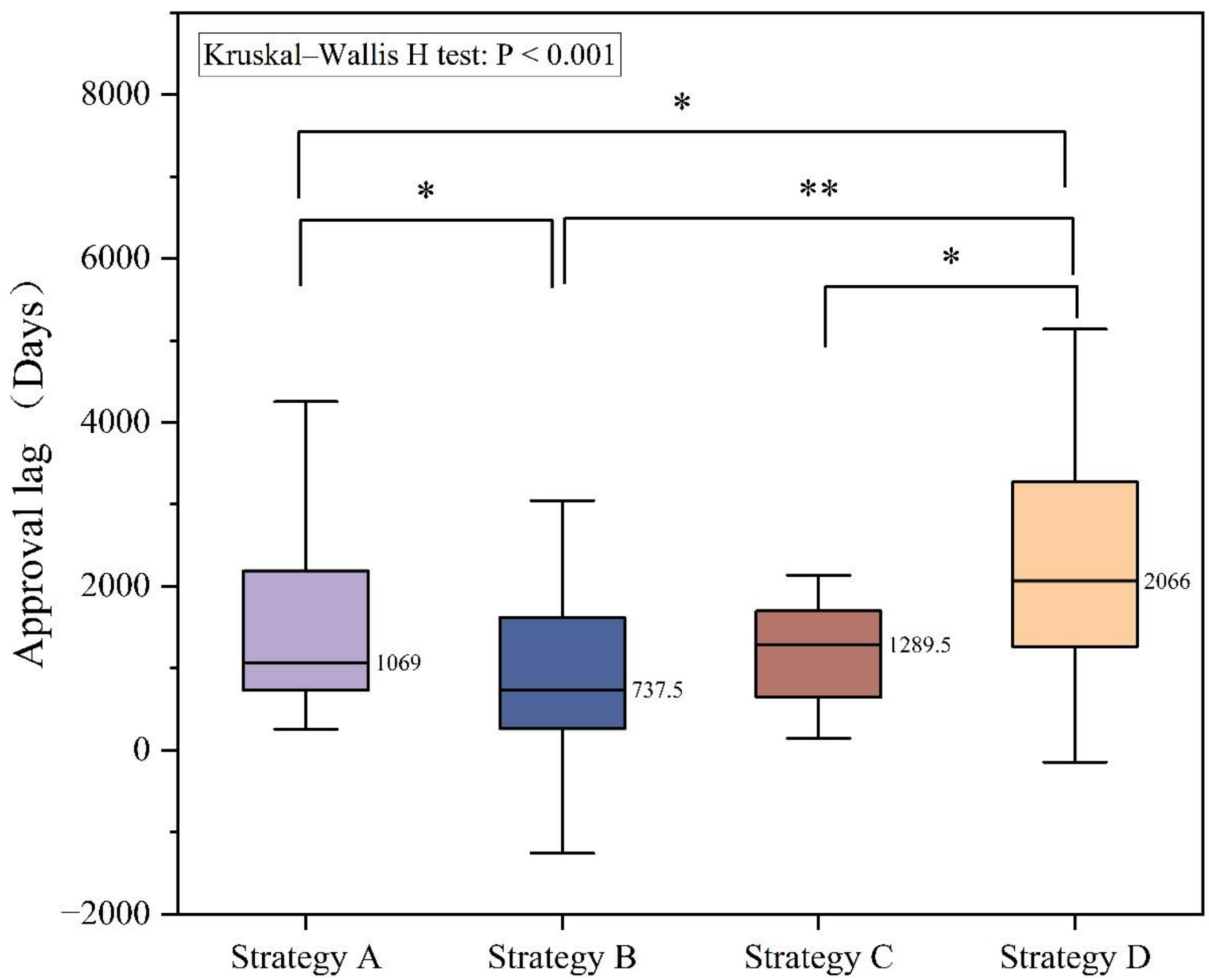

Note：*Adjusted P < 0.05; **adjusted P < 0.001.

Supplementary Figure S7 Approval lag across R&D strategies for overseas new drugs approved in China.

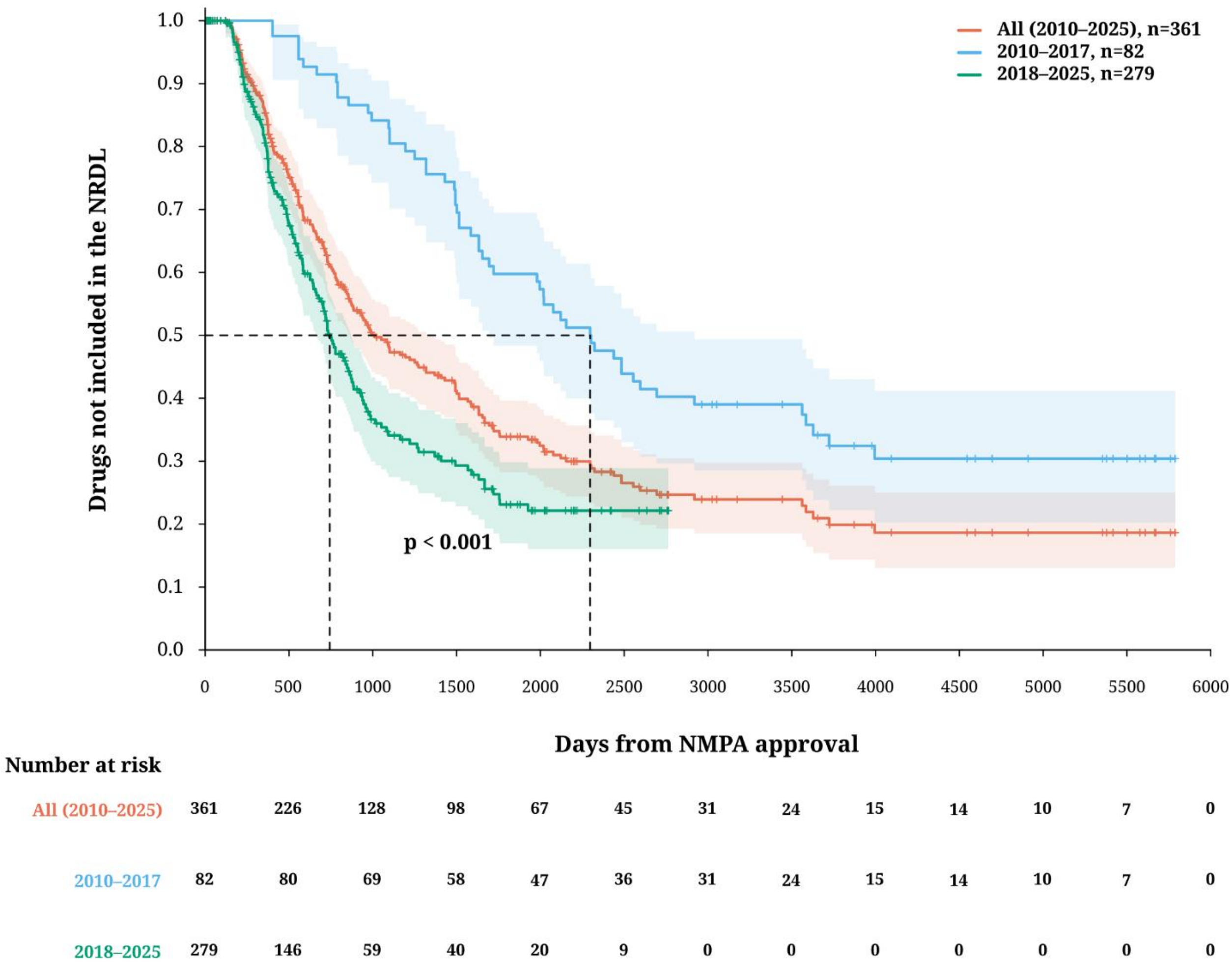

Supplementary Figure S8 Kaplan–Meier curves of time to reimbursement by NMPA approval period (2010–2017 vs. 2018–2025).

1.2 Supplementary Tables

Supplementary Table S1 Evolution of the regulatory reforms for overseas new drugs in China.

Initiation year	Policy document	Department	Measures
2007	Provisions for Drug Registration 2007	SFDA	• Require drugs included in MRCT in China to have been approved overseas or to have entered Phase II/III clinical development overseas, and require supplementary clinical trials involving Chinese patients for marketing authorization.
2015	Guidance on Multi-regional Clinical Trials (provisional)	CFDA	• Provide guidance on the application, implementation, and management of MRCT in China
2015	Opinions on Reforming the Review and Approval System for Drugs and Medical Devices	State Council	• Permit clinical trials of new drugs to be conducted simultaneously in China and overseas
2016	Opinions on Implementing a Prioritized Review System to Avoid Drug Review Backlogs	CFDA	• Allow new drugs with obvious clinical value to be granted priority review
2017	Decision on Adjusting Matters Concerning the Administration of Imported Drug Registration	CFDA	• Allow Phase I international multicenter clinical trials to be conducted simultaneously in China and abroad<br>• Allow submission for marketing based directly on data from multi-regional clinical trials<br>• No longer require prior approval in the country of origin.
2018	Technical Guiding Principles for the Acceptance of Overseas Clinical Trial Data of Drugs	NMPA	• Clarify the applicable scope, basic principles, integrity requirements, technical requirements for data submission, and the extent of acceptance for overseas clinical trial data
2018	Announcement on Optimizing the Review and Approval of Clinical Trials for Innovative Drugs	NMPA	• A 60-working-day implied approval period is implemented for clinical trial applications (IND) of innovative drugs
2018	Announcement on Matters Concerning the Evaluation, Examination and Approval of Foreign New Drugs Urgently Needed in Clinical Treatment	NMPA	• A fast-track review and approval channel is established for urgently needed overseas drugs

2020	Provisions for Drug Registration 2020	NMPA	• Formally consolidate expedited programs into the drug registration framework, including PR, CA and BTD
2020	Clinical Technical Requirements for Drugs Marketed Overseas but Not Marketed in China	NMPA	• Determine clinical trial requirements based on clinical data in different scenarios of imported drugs
2024	Interim Provisions on the Management of Designated Domestic Responsible Persons by Overseas Marketing Authorization Holders	NMPA	• Require overseas marketing authorization holders (MAHs) to designate a domestic responsible person with joint liability, and clarify the person's obligations and compliance responsibilities.
2025	Announcement on Matters Concerning the Importation of Pre-Approval Commercial-Scale Batch Products of Overseas-Marketed Drugs	NMPA	• Allow the importation and marketing of qualifying pre-approval commercial-scale batches of overseas-marketed drugs after approval in China, thereby facilitating earlier post-approval supply.
2025	Circular on the Pilot Program for Optimizing the Review and Approval Procedures for Supplemental Applications of Overseas-manufactured Drug	NMPA	• Optimize the review and approval of supplemental applications for overseas-manufactured chemical drugs, compressing the review time to 60 working days
2026	Announcement of the National Medical Products Administration on Matters Concerning Further Optimizing Review and Approval of Overseas-Marketed Drugs in Clinical Urgent Need	NMPA	• Encourage domestic applications for overseas-marketed originator and generic drugs in urgent clinical need, and allow eligible products to be included in the priority review pathway
2026	Announcement on Issuing the Implementation Measures for the Protection of Drug Trial Data	NMPA	• Grant a data protection period of up to 6 years for overseas new drugs

Note: CFDA, China Food and Drug Administration; NMPA, National Medical Products Administration; SFDA, State Food and Drug Administration.

Supplementary Table S2 Key features of China's approval procedure for UNODs.

Program	Approval procedure for urgently needed overseas drugs (UNODs)
**Time issued**	2018
**Objective**	Speeding up the entry of overseas new drugs urgently needed in clinical practice to China
**Qualifying criteria**	•Drugs for the treatment of rare diseases<br>• Drugs for the prevention and treatment of serious life-threatening diseases for which no effective treatment or prevention methods are available<br>• Drugs for the prevention and treatment of serious life-threatening diseases with obvious clinical advantages
**Application stage**	After completing communication and reaching consensus at meetings, formal NDA submission
**Advantages**	•Flexibility in the standard of efficacy and safety evidence<br>•Applicability for priority review<br>•Chance of exemption from domestic clinical trials

Supplementary Table S3 Representative borderline cases in R&D strategy classification.

Drug	Borderline issue	Evidence from CDE review documents	Final classification and rationale
Atezolizumab	Whether the China expansion cohort should be considered part of the global MRCT or as a separate China-specific supplementary clinical study.	The CDE review report stated that the core clinical evidence supporting the Chinese marketing application consisted of the global IMpower133 trial and its China expansion cohort. IMpower133 was an international multicenter phase I/III trial. After completion of global enrollment, approximately 100 additional Chinese patients were enrolled to assess the consistency of treatment benefit between the Chinese and global populations.	Strategy B. Although additional Chinese patients were enrolled after completion of the main global enrollment, the China expansion cohort remained part of the IMpower133 global clinical development program and was used to evaluate consistency with the global population rather than as an independent local confirmatory trial. Therefore, the drug was classified as Strategy B.
Crovalimab	Whether the drug should be classified as an MRCT-based development strategy or as a strategy based on overseas evidence supplemented by clinical evidence generated through newly conducted clinical trials in China.	The CDE review report indicated that the development program comprised early-phase studies conducted overseas and a clinical study conducted in China using patients' historical treatment data as the comparator, which served as the pivotal study for registration. The global phase III study was subsequently submitted as confirmatory evidence during regulatory review.	Strategy D. The pivotal clinical evidence for Chinese registration was generated through a separately conducted clinical study in China, while the global phase III study was submitted later as confirmatory evidence during regulatory review. Therefore, the development pathway was classified as Strategy D rather than an MRCT-based strategy.

Supplementary Table S4 Sensitivity analysis of approval lag between the pre- and post-reform periods using EMA and PMDA approvals as alternative comparators.

Comparator	Total, n	2010–2017, n	Approval lag, median (IQR), days	2018–2025, n	Approval lag, median (IQR), days	P value
EMA	300	65	1304.0 (886.5–2322.5)	235	986.0 (299.0–2068.0)	P=0.016
PMDA	286	71	1025.0 (343.0–1783.0)	215	774.0 (127.0–1775.0)	P=0.276

Note: Approval lag was defined as the interval between EMA or PMDA approval and NMPA approval. Negative values were retained and indicate earlier approval in China. Only drugs with matched approval records in the respective jurisdiction were included. P values were calculated using the Mann–Whitney U test.

Supplementary Table S5 Distribution of approval lag in China compared with the United States across R&D strategies.

Approval lag category compared with US approval	n (%)			
	Strategy A (n=83)	Strategy B (n=98)	Strategy C (n=22)	Strategy D (n=48)
Earlier approval in China	0 (0)	5 (5.1)	0 (0)	1 (2.1)
≤1 year after US approval	2 (2.4)	27 (27.6)	3 (13.6)	0 (0)
>1 year to ≤2 years after US approval	18 (21.7)	16 (16.3)	3 (13.6)	6 (12.5)
>2 years to ≤ 3 years after US approval	23 (27.7)	10 (10.2)	4 (18.2)	3 (6.3)
>3 years after US approval	40 (48.2)	40 (40.8)	12 (54.5)	38 (79.2)
P value	P=0.001			

Supplementary Table S6 Sensitivity analysis of factors associated with NRDL inclusion using an alternative period-specific definition.

Variable	N	%	OR	95% CI	P value
**Drug class**					
Chemicals	250	69.3	Reference		
Biologicals	111	30.7	0.464	0.279-0.772	P=0.003
**Approval year**[a]					
Pre-reform period	82	22.7	Reference		
Post-reform period	279	77.3	2.923	1.639-5.213	P<0.001

**Therapeutic area**					
Non-oncology	262	72.6	Reference		
Oncology	99	27.4	1.551	0.874-2.752	P=0.134
**Innovativeness**					
Original drug	328	90.9	Reference		
Innovative drug	33	9.1	0.953	0.410-2.219	P=0.912
**Rare disease**					
No	273	75.6	Reference		
Yes	88	24.4	0.576	0.320-1.039	P=0.067
**UNOD**[b]					
No	317	87.8	Reference		
Yes	44	12.2	3.174	1.416-7.115	P=0.005
**Target population**					
Adults only	289	80.1	Reference		
Pediatric patients included[c]	72	19.9	0.538	0.292-0.993	P=0.047
PR[d]					
No	189	52.3	Reference		
Yes	172	47.7	1.219	0.722-2.055	P=0.459
CA[d]					
No	311	86.1	Reference		
Yes	50	13.9	0.421	0.199-0.889	P=0.023
BTD[d]					

No	343	95.0	Reference		
Yes	18	5.0	0.943	0.307-2.890	P=0.918

Note: Under the alternative period-specific definition, drugs were classified as included in the NRDL only if their first NRDL inclusion occurred within the same analysis period as their initial NMPA approval (2010–2017 or 2018–2025). All other drugs were classified as not included.
[a]Approval year refers to the year of initial approval by the NMPA. [b]UNOD refers to an overseas new drug included in any of the lists issued by the National Medical Products Administration. [c]Pediatric patients included refers to drugs indicated either exclusively for pediatric patients or for both adult and pediatric patients. [d]PR, Priority Review; CA, Conditional Approval; BTD, Breakthrough Therapy Designation.

Supplementary Table S7 Approval lag and reimbursement outcomes stratified by expedited regulatory pathway.

**Expedited pathway**	**Total, n**	**FDA-matched, n**	**Approval lag, median (IQR), days**	**NRDL inclusion, n (%)**	**Time to reimbursement, median (IQR), days**
PR	172	160	990.0 (489.0–1585.8)	104/172 (60.5)	555.5 (356.5–854.8)
CA	50	48	801.5 (490.3–1497.5)	22/50 (44.0)	687.5 (437.5–970.8)
BTD	18	18	171.5 (113.5–610.0)	9/18 (50.0)	345.0 (218.0–448.0)

Note: PR, Priority Review; CA, Conditional Approval; BTD, Breakthrough Therapy Designation. Drugs with multiple designations were included in each applicable category. Approval lag analyses included only drugs with corresponding FDA approval records. NRDL inclusion status was assessed as of January 1, 2026.
